# Impact of Climate Change on Health and Well-Being of People in Hindu Kush Himalayan Region: A Narrative Review

**DOI:** 10.3389/fphys.2021.651189

**Published:** 2021-08-06

**Authors:** Meghnath Dhimal, Dinesh Bhandari, Mandira Lamichhane Dhimal, Naviya Kafle, Prajjwal Pyakurel, Narayan Mahotra, Saeed Akhtar, Tariq Ismail, Ramesh C. Dhiman, David A. Groneberg, Uttam Babu Shrestha, Ruth Müller

**Affiliations:** ^1^Nepal Health Research Council, Kathmandu, Nepal; ^2^Global Institute for Interdisciplinary Studies, Lalitpur, Nepal; ^3^School of Public Health, The University of Adelaide, Adelaide, SA, Australia; ^4^Policy Research Institute, Kathmandu, Nepal; ^5^Department of Community Medicine, BP Koirala Institute of Health Sciences, Dharan, Nepal; ^6^Institute of Medicine, Tribhuvan University, Kathmandu, Nepal; ^7^Institute of Food Science and Nutrition, Bahauddin Zakariya University, Multan, Pakistan; ^8^ICMR-National Institute of Malaria Research, New Delhi, India; ^9^Institute of Occupational, Social and Environmental Medicine, Goethe University, Frankfurt am Main, Germany; ^10^Institute of Tropical Medicine, Antwerp, Belgium

**Keywords:** climate change, health, mountain, non-communicable disease, infectious disease, mental health, gender, disasters

## Abstract

Climate change and variability affect virtually everyone and every region of the world but the effects are nowhere more prominent than in mountain regions and people living therein. The Hindu Kush Himalayan (HKH) region is a vast expanse encompassing 18% of the world’s mountainous area. Sprawling over 4.3 million km^2^, the HKH region occupies areas of eight countries namely Nepal, Bhutan, Afghanistan, Bangladesh, China, India, Myanmar, and Pakistan. The HKH region is warming at a rate higher than the global average and precipitation has also increased significantly over the last 6 decades along with increased frequency and intensity of some extreme events. Changes in temperature and precipitation have affected and will like to affect the climate-dependent sectors such as hydrology, agriculture, biodiversity, and human health. This paper aims to document how climate change has impacted and will impact, health and well-being of the people in the HKH region and offers adaptation and mitigation measures to reduce the impacts of climate change on health and well-being of the people. In the HKH region, climate change boosts infectious diseases, non-communicable diseases (NCDs), malnutrition, and injuries. Hence, climate change adaptation and mitigation measures are needed urgently to safeguard vulnerable populations residing in the HKH region.

## Introduction

**Figure 1:**
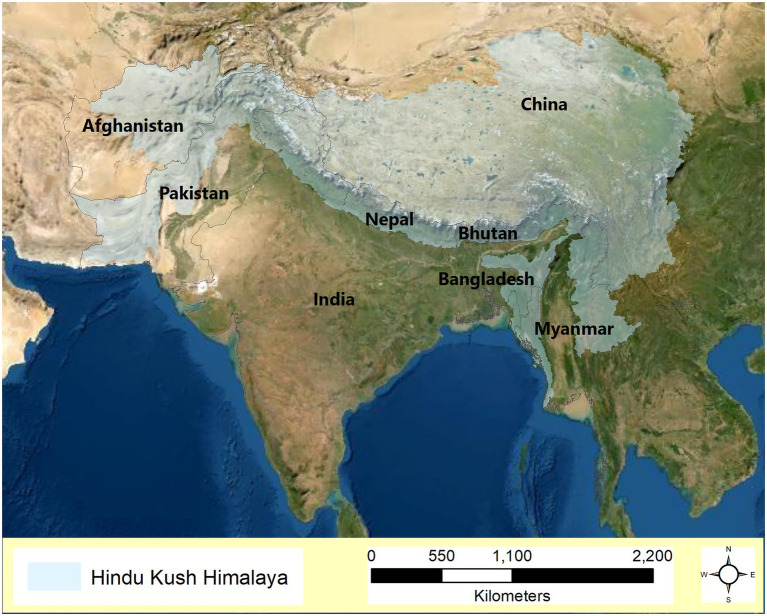
Map showing Hindu kush Himalayan region.

The consequential social and environmental changes due to globalization, interconnectedness, travel, trade, and an emphasis on economic and political supremacy are unprecedented. Disruption in the natural biogeochemical cycles as a result of anthropogenic climate change is now gradually approaching the safety limit for all life forms on Earth (McMichael, 2013). Given the alteration in the Planet’s life-sustaining systems, climate change has been identified as the greatest global health threat of the 21st century (Watts et al., 2018). Hindu Kush Himalayan (HKH) region (Figure 1), the largest mountain system on earth and the origin of 10 major river basins that support almost 2 billion people, is one of the world’s most vulnerable ecosystems and is highly susceptible to the impact of climate change (Shrestha et al., 2012; Pandit et al., 2014; Wester et al., 2019). The rate of warming in this region (0.06°C per year, estimated using the baseline data spanning 25 years prior to the year 2006) has been reported to be greater than the global average warming rate (Shrestha et al., 2012).

An assessment of the physical and biological environment in the HKH region shows a catastrophic increase in social and economic vulnerability of its inhabitants, which consequently threatens the physical and mental well-being of the people living in this region (Wester et al., 2019). Variations in temperature and precipitation patterns, through diverse pathways and complex mechanisms, impact adversely the well-being of HKH residents. Health impacts of climate change can be experienced in the form of psychological impacts such as increased suicide rate, post-traumatic disorders following extreme weather events, loss of relationship and identity, substance abuse, and feelings of hopelessness and/or physiological impacts such as rise in climate-sensitive infectious diseases, morbidity and mortality due to heatwaves, increase in non-communicable diseases (NCDs), injury, and illness related to extreme weather events, malnutrition and stunting, respiratory problems due to increased allergens and pollutants (WHO, 2009; IPCC, 2014; Woodward et al., 2014; Hayes and Poland, 2018). While evidence on both, the psychological and physical impacts of climate change on the inhabitants of coastal regions, lowlands, and plains across the globe is abundant, health impacts on the residents of high mountains as in the HKH remain relatively unexplored. Hence, in this narrative review, we synthesize evidence on the impact of climate change on physical and mental well-being of the people living in the HKH and discuss the plausible health impacts on the residents that may be attributed to climate change.

## Materials and Methods

This study is based on a narrative review. We chose to perform a narrative review due to (i) concerns that the systematic review process will omit many studies based on strict inclusion/exclusion criteria; (ii) there are limited publications on climate change and health in the HKH region; and (iii) the broad scope of the present review, which is unsuitable if we use the systematic process for. A literature review was performed using keywords search (“climate change,” “health,” “HKH region,” “infectious diseases,” “gender,” “NCDs,” “mountain biodiversity,” and “disaster”) in PubMed and Google Scholar databases. The reference list of relevant articles was also scanned for their potential inclusion. These two databases were chosen because PubMed remains an optimal tool in public health and biomedical research, as such, the chances of missing relevant articles using PubMed search are rare. These databases are publicly accessible and without a paywall. To minimize omission, we additionally used Google Scholar.

## Results

### Vector-Borne Diseases in the HKH Region

Climate change that promotes the mobility of humans and animals including vectors and hosts of different diseases is considered a driver of emerging infectious diseases (IPCC, 2014; Woodward et al., 2014; Bongaarts, 2019). It has threatened to undermine public health advances achieved over the last several decades. Concerted global efforts over the past 4 decades have reduced the global burden of malaria but morbidity and mortality associated with other vector borne diseases, especially dengue, remained on the rise, particularly in the global south (World Health Organization, 2012). More recently, the lancet commission on climate change and health 2019 revealed that suitability of disease transmission (malaria and dengue) by mosquito vectors has increased over the past few years (Watts et al., 2019). This deduction by the leading body of public health researchers specialized in climate change and health effects is underpinned by the geographic expansion of disease vectors and subsequently confirmed cases of vector-borne diseases (VBDs) in the high mountain regions of the world including HKH (Bouma et al., 1996; Bi et al., 2013; Dhimal et al., 2015a,b, 2018, 2021; Dhiman et al., 2019). Studies suggest that the increasing temperature and frequency of extreme rainfall events in high mountains of South Asia is now more conducive for the survival and reproduction of disease-carrying vectors (Dhiman et al., 2011; Dhimal et al., 2015b, 2021; Sarkar et al., 2019).

Vector-borne diseases are among the most studied climate sensitive infectious diseases; hence, mechanisms behind their interaction with climatic factors and the consequential risk of infection are well established (Campbell-Lendrum et al., 2015). Epidemic potential of VBD, in general, has been estimated to increase with the increase in temperature (Patz et al., 1998), which holds equally true for the HKH region. Likewise, increased rainfall may affect the reproduction rate of disease vectors in the HKH region. Furthermore, climate change also facilitates the rapid expansion of the host range and microbial capacity to colonize new hosts (Brooks et al., 2019). While the host factors related to the vulnerability of people living in HKH may be comparable to people living in other parts of the globe; the environmental factors such as disproportionate increase in temperature in high mountains, reduced snowfall, extreme weather events such as heavy rainfall, increased agricultural and farming activities in mountain slopes, and increased accessibility and mobility of people are unique to HKH region (Ebi et al., 2007). These changes observed in the HKH region over the last few decades are likely to increase the risk of VBDs transmission in the context of a changing climate (Dhimal et al., 2018).

Climate change is implicated in the geographical expansion of autochthonous cases of VBDs to non-endemics areas of high mountains in Nepal (Dhimal et al., 2015a). The expanded spatial distribution and increased incidence of chikungunya and dengue over the last decades in the HKH region have been associated with climate change (Phuyal et al., 2020; Dhimal et al., 2021). Increasing number of confirmed cases of Japanese encephalitis in Himalayan highlands, which were previously confined to the lower southern plains, has been linked with climate change (Baylis et al., 2016). Likewise, an entomological survey in the higher mountains of central and eastern Nepal reported the presence of dengue vectors *Aedes aegypti* and *A. albopictus*, vector of Japanese encephalitis *Culex tritrinorinchoes*, malaria vectors *Anopheles fluviatilis*, *A. annularis*, and *A. maculatus* complex as well as lymphatic filariasis vector *C. quinquefasciatus* (Dhimal et al., 2014a, 2015b). Given the establishment of human biting *Aedes* mosquitos, the HKH region has been identified as a potential threat zone for outbreaks of Zika virus infection (Dhimal et al., 2018). A model-based study projecting the future risk of dengue virus infection in Nepal under different climate scenarios as denoted by Representative Concentration Pathways (RCP 2.6, RCP 6.0, and RCP 8.5) predicts geographic expansion of dengue virus infection hotspot to the higher elevation regions by 2050–2070 (Acharya et al., 2018). Increased cases of malaria with the rise in temperature and increased rainfall has also been reported from highland regions of Bangladesh, Pakistan, Bhutan, Nepal, and India (Bouma et al., 1996; Haque et al., 2010; Dhiman et al., 2011, 2019; Dhimal et al., 2014b; Wangdi et al., 2020), as shown in Table 1. Sarkar et al. (2019) simulated the future implications of climate change on malaria transmission in India and predicted western Himalayan states to be a potential focus for *Plasmodium vivax* malaria transmission. Different climate scenarios (RCP 2.6, RCP 6.0, and RCP 8.5) anticipate similar geographic expansion of malaria vectors in the southern Highlands of China by 2030–2050 (Ren et al., 2016). A recent review on climate change and infectious diseases from China has also revealed an association between increased temperature and malaria cases in southwestern highland regions of China including Tibet (Yi et al., 2019).

**Table 1:** Epidemiological evidence on the impacts of climate change on vector borne disease in Hindu Kush Himalayan (HKH).

Study region	Study period	Disease	Findings	References
Bhutan	2016–2019	Dengue	Dengue cases increased by 63% (95% CI: 49, 77%) for a 1°C increase in maximum temperature.	Tsheten et al., 2020
India (including the northern Himalayas)	N/A	Malaria	Areas of the western Himalayan states are likely to have new foci for malaria transmission.	Sarkar et al., 2019
China	2005–2014	Malaria	The distribution of malaria is expected to increase in most regions regardless of the climate scenarios.	Hundessa et al., 2018
Nepal (including high Himalayan region)	N/A	Dengue	Under the different climate change scenarios, the vulnerability of dengue in Nepal will be shifted toward higher elevation with varied magnitude and spatial patterns.	Acharya et al., 2018
China (multi cities including mountainous provinces)	2005–2014	Hemorrhagic fever with renal syndrome	A 1°C increase in maximum temperature resulted in 1.6% increase in HFRS. Similarly, 1mm increase of weekly precipitation was associated with 0.2% increase in HFRS.	Xiang et al., 2018
China (Yunnan province)	2005–2010	Malaria	A 1°C increase in minimum temperature was associated with increased risk (RR = 1.03; 95% CI, 1.01, 1.05) of *P. vivax* malaria at lag 7 weeks.	Bi et al., 2013
Bangladesh (Hilly district)	2009–2012	Malaria	Malaria incidence was positively associated with rainfall (*R*^2^ = 0.252; *p* = 0.007) and minimum temperature (*R*^2^ = 0.203; *p* = 0.016).	Ahmed et al., 2013
Nepal (Morang and Kailali Districts)	2007–2011	Malaria	A 1°C increase in minimum and mean temperatures increased malaria incidence by 27% (RR = 1.27, 95% CI = 1.12–1.45) and 25% (RR = 1.25, 95% CI = 1.11–1.43), respectively.	Dhimal et al., 2014b

### Water- and Food-Borne Diseases in the HKH Region

Water- and food-borne diseases are mainly transmitted through consumption of contaminated water and food – as a consequence of shortage of clean drinking water; lack of hygienic practices, and unavailability of proper food storage facilities, all of which are pervasive in the remote areas of HKH. The classical clinical manifestation or outcome of water- and food-borne diseases is acute diarrhea, which remains a leading cause of death among children below age 5 years (Troeger et al., 2017). An increasing body of evidence suggests that transmission and onset of water- and food-borne illness is highly influenced by climate variabilities in temperature, humidity, and rainfall, periodic events like El Nino, and climate-induced disasters like floods (Checkley et al., 2000; Singh et al., 2001; Liu et al., 2017). Given the higher prevalence of childhood diarrhea and a higher mortality rate among the children of rural mountains in South Asia including countries of the HKH region (Reiner et al., 2020), increased prevalence of diarrheal disease among the people living in the HHK region in the context of changing climate is a major concern. All the means and mechanisms of water- and food-borne diseases transmission are plausible in the HKH region that makes this region a hotspot for diarrheal diseases outbreak under the influence of future climate change.

Despite the higher vulnerability, limited evidence is available on the impacts of climate change in water- and food-borne illness in the HKH region due to the lack of health surveillance data available at the local scale. However, some localized studies identified linkages between climate change and water and food borne diseases. For example, Wangdi and Clements (2017) analyzed nationwide diarrheal datasets in Bhutan, spanning a period of 1 decade and reported that 0.6 and 5% increase in risk for diarrheal incidence per every 1°C rise in maximum temperature and 1 mm increase in rainfall, respectively (Table 2). A study from Pakistan reported that increased temperature and climate induced-flooding events had exacerbated water borne diseases such as cholera and infective gastroenteritis in the mountainous province of Baluchistan (Malik et al., 2012).

**Table 2:** Epidemiological evidence on the impacts of climate change on food- and water-borne diarrhea in HKH region.

Study region	Study period	Disease	Findings	References
Afghanistan	2010–2016	All cause diarrhea	For every 1°C increase in mean daily temperature incidence of diarrhea increased by 0.70% (95% CI: 0.67, 0.73%).	Anwar et al., 2019
Nepal (3 ecological belts including Himalayan region)	2002–2014	All cause diarrhea	For every 1°C increase in average temperature the risk of diarrhea incidence increased by 0.85–5.05% (highest impact felt in mountains). The overall effect for Nepal was found to be 4.39% (95% CI: 3.95–4.85).	Dhimal et al., 2016
Bhutan (national study including high mountains)	2003–2013	All cause diarrhea	For every 1°C increase in maximum temperature, incidence of diarrhea increased by 0.6% (95% CI: 0.5–0.6%).	Wangdi and Clements, 2017
South China (mountainous province)	2004–2010	Bacillary dysentery	Compared with the YLDs in 2010, increasing flood events may lead to a 4.0% increase in the YLDs for bacillary dysentery by 2020 and 8.0% increase by 2030 in Guangxi, China.	Liu et al., 2017
North India	Future projection	All cause diarrhea	A 1.8°C increase in future temperature in the Ganges basin is expected to increase the burden of diarrhea in north India by 10% by the 2040s.	Moors et al., 2013
Balochistan, Pakistan (mountainous region)		Cholera	Increase in temperature and flood has led to increase in the burden of cholera and other gastro-enteric infections causing diarrhea.	Malik et al., 2012

Likewise, national and subnational level assessments of the impacts of climate change on diarrheal diseases in Nepal reported a greater impact of climate change on diarrheal diseases incidence among the residents of mountain regions compared to lowland (Dhimal et al., 2016). The study reported an overall increased risk of diarrhea (5.05% per 1°C rise in average temperature) for the mountainous regions, compared to non-mountainous parts of the country. Similarly, for a mountainous region of Southern China, climate-induced flooding events are projected to increase the years lived with disability from bacillary dysentery by up to 8% by the 2030 compared to 2010 (Liu et al., 2017). Moors et al. (2013) used regional climate models to project the future burden of waterborne diarrhea in Ganges basin of Northern India and predicted 13.1% increase in diarrhea cases attributable to climate change by the year 2040 in comparison to the late 1990s. So far, the only study published from Afghanistan has reported a positive association between mean daily temperature and nationwide diarrhea incidence, with 0.70 and 4.79% increased risk of diarrhea for every 1°C rise in mean daily temperature and 0.01 unit change in aridity index, respectively (Anwar et al., 2019).

Several biological and physical mechanisms have been proposed to illustrate potential mechanism by which climate change is likely to enhance transmission of water- and food-borne illnesses (Levy et al., 2016; Bhandari et al., 2020a,b). Apart from the general mechanisms common to most geographical regions, a plausible mechanism possibly unique to the HKH region is the contamination of drinking water sources due to the disruptions of sanitary and sewage systems during extreme weather events such as floods resulting from the outburst of glacial lakes (Ebi et al., 2007). Endowed with the world’s tallest mountains and famous trekking routes, the HKH region is a popular tourist destination among mountaineers and trekkers. Climate change is likely to increase the warm days conducive for trekking and mountain expeditions, which in turn may overcrowd the mountains. Open defecation or improper disposal of sludge and excreta during trekking or mountain expedition may contaminate the springs and water sources (Associated Press, 2019), resulting in outbreaks of waterborne diseases. Likewise, increased frequency of travelers in the mountains – in an already compromised ecosystem with limited resources to sustain the resident population – may introduce new strains of entero-pathogens such as Cholera, which carries epidemic potential. Introduction of epidemic strains of entero-pathogens by a foreigner in an already vulnerable ecosystem was previously recorded in Haiti during the aftermath of the 2010 earthquake (Orata et al., 2014).

#### Adaptation and Mitigation Measures for Climate Sensitive Infectious Diseases

The HKH region bears a significant proportion of global infectious diseases burden due to multiple risk factors including rapid urbanization and increased land use change, high degree of air pollution, poor sanitation, a poorly developed healthcare system, and low socio-economic status of people living in this region (Laxminarayan et al., 2017). Hence, the adaptation and mitigation measures for both VBDs and food- and water-borne diseases should consider non-climatic determinants such as population growth, urbanization, land use change, socio-economic developments, in addition to the climatic determinants (Ebi et al., 2013). Adaptation measures should be designed to prevent population exposure to climate hazards and extreme weather events considering the projected increase in future population as well as projected disease burden attributable to climate change. It is imperative that public health infrastructures in the HKH region be strengthened to support the establishment of early warning systems for epidemic-prone and climate-sensitive infectious diseases (Bhandari et al., 2020c). The establishment of a regional disease intelligence collection system or an integrated surveillance system, as well as trans-border collaboration of interdisciplinary experts within the HKH region, is necessary to mitigate the impact of climate change on infectious diseases.

### Food and Water Insecurity and Malnutrition in the HKK Region

Diverse policy responses to climate change have been initiated worldwide including in the mountainous regions. Global debates and dialogs weighing the consequences of climate change in these regions were also realized at Sendai Framework for Disaster Risk Reduction 2015 and the Paris Agreement 2015 of the United Nations Framework Convention on Climate Change (Wester et al., 2019). Similarly, climate change and its impact on human livelihood are now an integral part of the development plans and programs of governments in the HKH region (Ahmed et al., 2019). Despite these initiatives, climate change has already impacted food, water, and nutrition security of the region. Due to the considerable variations in agro-ecological and livelihood resources across the HKH region, disproportionate impacts of climate change on these sectors were observed (Hussain et al., 2016; Merrey et al., 2018). Unprecedented climate changes including increased temperature, year-to-year changing precipitation patterns, frequent floods combined with other drivers such as deforestation and forest degradation, and commercialization of productive agriculture lands are deleteriously affecting agriculture and food security in the HKK region (Nautiyal et al., 2007).

The direct impact of climate change on agricultural productivity accompanied by increased risk of vector–borne infectious disease was reported in a household survey conducted in Rakaposhi valley of Gilgit – Baltistan, Pakistan (Bhatta et al., 2019). Ground water extraction to cope with the uncertainties of surface water availability in the HKH region in anticipation of increased energy demand and severe losses to scarce water resources (Rasul and Sharma, 2016). High incidence of food and nutritional insecurity is prevailing in western and far-western mountainous regions of Nepal, Afghanistan, Chin state in Myanmar, Baluchistan province of Pakistan, and Meghalaya state of India. Climate-induced changes like floods, droughts, landslides, livestock diseases, and increased biological invasions including crop pests have led to decreased food production and farm income for the local HKH communities (Hussain et al., 2016; Shrestha and Shrestha, 2019).

The state of food and nutrition security in the HKH region is not promising especially in remote mountain areas owing to a high incidence of natural calamities, physical constraints to better agricultural productivity, poor infrastructure, and high cost of transportation, and limited access to food markets (Huddleston et al., 2003; Ward et al., 2013). In addition, decreased productivity in agriculture with the changing climate exacerbates food insecurity and malnutrition in the region. Evidently, the causes of food security and malnutrition differ in mountains and plains (Rasul et al., 2019). Drought affects livelihoods in the HKH region by engendering food insecurity through reduced water availability for both agriculture and rangeland production (Joshi et al., 2013; Rasul et al., 2014).

In the face of extensive malnutrition, food insecurity and dampened health care systems in the HKH region, no concerted plans are put in place focusing on enhancing food production and increasing household incomes. For example, one third to half of the children below 5 years of age from the HKH region suffer from stunting and wasting. Climate change and deteriorating agro-ecological environment turn up to be the most significant contributing factors toward food and nutrition insecurity in the region (Rasul et al., 2018). For example, almost two-thirds of Baluchistan and the Federally Administered Tribal Areas (FATA) of Pakistan are extremely food insecure by dint of limited income to purchase sufficient food by the local population (Hussain and Routray, 2012). Myriad challenges worsening food security and nutrition in the HKH region encompass debilitated health care systems, unavailability of clean drinking water, insufficient sanitation, unsafe food, limited knowledge of nutrition at household level, and inability to empower women (Rasul et al., 2019). Climate change has further exacerbated the existing food security challenges.

#### Adaptation and Mitigation Measures for Food, Water Insecurity, and Malnutrition in the HKK Region

Increased climate variability is already affecting water availability, provision of ecosystem services, agricultural production, and people’s livelihood in the HKH region (Mishra et al., 2019). Furthermore, the high mountains are poorly served by life-saving and livelihood-supporting infrastructure. Hence, increasing access to climate information and support services is of utmost importance. Strengthening the institutional links may empower farmers to adopt technology that can contribute to increased adaptive capacity. More importantly, mitigation and adaptation to climate changes in the HKH region require a concerted approach at global, national, and sub-national levels. These strategies must entail efforts to improve sustainable production for the purpose of ensuring food and nutritional security in the region (Mishra et al., 2019). About 50% of the population of regions, population suffers from malnutrition, and women and children suffer more. Therefore, ending hunger and achieving food and nutrition security – as articulated in the Sustainable Development Goals (SDGs) is an urgent need for countries and the developmental partners (Rasul et al., 2019). Finally, food insecurity has the greatest impact on those people in the HKH region who are socially, culturally, economically, or otherwise marginalized. Hence, achieving improved food security in the face of climate change requires: bridging the knowledge gaps about food production systems, targeting and increasing involvement of younger generations in farming, supporting greater diversity in small-scale farming, developing more gender-sensitive farming approaches, strengthening education, and building effective networks for knowledge sharing, and integrating food security development goals in policies that address climate change adaptation and mitigation within the broader framework of SDGs (Kurvits et al., 2014).

### Non-communicable Diseases and Mental Health in the HKH Region

#### Anticipated Effects of Climate Change on Non-communicable Diseases and Mental Health

Climate change will exacerbate the incidence of NCDs including cardiovascular disease, cancer, respiratory health, mental disorder, injuries, malnutrition (Kjellstrom et al., 2010; Friel et al., 2011; Campbell-Lendrum and Prüss-Ustün, 2019; Nugent and Fottrell, 2019), and mental health (Berry et al., 2010; Bourque and Cunsolo Willox, 2014; Hayes et al., 2018; Cianconi et al., 2020). Studies show that exposure to extreme temperature at either end of the tolerable range is associated with an increased risk of cardio-pulmonary mortality. The underlying physiological mechanism could be explained by direct link with increased blood pressure, viscosity, and heart rate for CVD and bronchoconstriction for pulmonary disease (McMichael et al., 2003). Temperature increase lowers agriculture production in the poorer tropics, and global food production will fall if the temperature warms by >3°C, again predisposing to NCDs (The Guardian, 2011). A wide range of risk factors for NCDs are strongly linked to environmental exposures – and to climate change; hence, the combination of climate change, air pollution, and NCDs is among the most serious threats to global health (Campbell-Lendrum and Prüss-Ustün, 2019). As global warming intensifies, incidences of severe heat waves, droughts, storms, and floods will become more frequent and severe, and will likely exacerbate the incidence of some NCDs, including cardiovascular disease, some cancers, respiratory health, mental disorders, injuries, and malnutrition globally (Friel et al., 2011). Additionally, increase in atmospheric temperature due to changing climate conditions will compromise outdoor air quality by increasing the production of tropospheric ozone. Climate change may increase air pollution thus augmenting the risk of cardiovascular disease through three main exposure pathways: directly *via* air pollution and extreme temperatures and indirectly *via* changes to dietary options (Friel et al., 2011).

A number of studies has been conducted to better understand the effects of climate change incidence and mortality of NCDs, specifically in the HKH region (Table 3). Majority of these studies have been conducted in China, and there are very few studies examining this relationship in other regions, signifying a gap in research.

**Table 3:** Epidemiological evidence on the impacts of climate change (increased in temperature) on non-communicable diseases (NCDs) and mental health in the HKH.

Study region	Study period	Disease	Findings	Study
China (184 cities including those from northern provinces)	2014–2017	Cardiovascular	For each 1°C increase in daily temperature, incidence of cardiovascular disease increased by 0.44% (0.32–0.55%).	Tian et al., 2019
China (rural villages from northern mountains)	2012–2015	Cardiovascular	Compared to low-risk temperature (17.3°C), the risk of cardiovascular disease was highest (RR: 1.28; CI: 1.11–1.48) at the 99th percentile.	Zhao et al., 2018
China (national study including alpine regions)	2012–2015	Hypertension	10°C decrease in ambient temperature was statistically associated 0.74 mmHg (95% CI: 0.69, 0.79) and 0.60 mmHg (95% CI: −0.63, −0.57) rise for Systolic Blood Pressure and Diastolic Blood Pressure, respectively.	Kang et al., 2020
India	Cross sectional 2011	Cardiovascular strain	The peak heart rate (HRp) was significantly higher in the air temperature (Ta) ranges of 31–33.5°C (*p* < 0.05) and 35–36°C (*p* < 0.001) than at 28–30°C.	Sahu et al., 2013
China	2007–2013	Diabetes	For each 1°C increase in daily mean temperature above the threshold of 31°C, mortality due to diabetes related cases increased by 30.5%.	Li et al., 2017
China	Cross sectional 2010	Mental health	Heat wave events with a lag period of 3 days were associated with an increased odds of hospitalization 3.178 (95% CI: 1.995–5.064) for mental health problems.	Liu et al., 2018

Besides NCDs, mountain communities are also at risk of climate-induced mental health challenges carried by increase in natural disasters such as drought (Cianconi et al., 2020). It is reported that many people exposed to climate or weather-related natural disasters experience stress and serious mental health consequences because of death of spouse or family members, resources, social support and social networks, or extensive relocation, and face post-traumatic stress disorder (PTSD), depression, and general anxiety, increased substance use or misuse, and suicidal thoughts (The US Global Climate Program, 2018). There are limited studies on impacts of climate change on NCDs and mental health in the HKH region; however, existing evidence highlighting the immense impacts of climate change on the health and well-being of HKH populations points to a great need to pay attention to the issue.

#### Adaptation and Mitigation Measures

The adaptation and mitigation strategies of climate change on NCD and Mental Health could be broadly classified into three sectors, namely, energy, municipal planning, and food and agriculture. In the energy sector, clean energy such as electricity could be generated which can reduce the use of biomass fuel. Additionally, improving home energy performance through efficient heating and cooling mechanisms will also reduce greenhouse gas emission leading to a decrease in indoor and outdoor pollution; hence, reduced the risk of NCDs. Additionally, municipal planning could be improved through developing road lanes reserved for walking and cycling, thereby lowering greenhouse gas emissions and building parks, promoting physical activity which reduces obesity and respiratory illness and diseases. Lastly, *food* cultivation could be improved by decreasing consumption of animal products and supporting new food harvesting approaches and rural employment, food system heterogeneity, and investment in urban farming (Friel et al., 2011).

### Climate Induced Disasters and Health in the Region

Climate change in the HKH region increases the frequency and magnitude of both “rapid-onset” hazards and “slow-onset” hazards (Eriksson et al., 2008). Floods, landslides, glacial lake outburst floods (GLOFs), flash floods, and debris flows are categorized as “rapid-onset” hazards, while droughts, heat waves, cold spells, and biological and hydrological changes are “slow-onset” hazards. The frequency and magnitude of GLOFs have increased along the region in recent decades, causing large social and economic damages (Eriksson et al., 2008).

Atmospheric warming in the Himalayan region causes glacial retreat and the formation of meltwater lakes, increasing the likelihood of disasters such as GLOFs. GLOFs are not only an environmental threat but also the cause of social and economic hardship for affected populations. The incidence of GLOFs is increasing in the HKH region. For instance, in the past 7 decades, 20 GLOF events have been recorded in the Himalayan region, which have caused the destruction of life and property, infrastructure as well as agricultural lands and forests. A report released by ICIMOD has mapped and classified more than 25,000 glacial lakes in the HKH region, of which 47 have been labeled potentially dangerous GLOF hazards (Bajracharya et al., 2020). Climate-induced heavy rainfall causes floods, landslides and extreme precipitation interrupts power, contaminates water sources, damages roads, and closes health centers all of which affect public health.

Flash floods destroy crops, directly affecting the supply chain of food, water, sanitation, and hygiene. Floods’ impacts are felt differently among gender and affect women disproportionately. For instance, the flood-related mortality was higher for girls (13.1 per 1,000) compared to boys (9.4 per 1,000), and higher for women (6.1 per 1,000) compared to men (4.1 per 1,000) during the severe flood in Sarlahi district in 1993 in Nepal (Pradhan et al., 2007). Moreover, poor and marginalized groups are more vulnerable to climate-induced disasters because of their settlement in river banks, flood plains and far away from major settlements in which mainly rich and socially advantaged groups reside. This in turn delays rescue and support operations during and after such disasters (Gurung and Bisht, 2014). These examples suggest that disasters will have the greatest impact on poor women and marginalized populations.

Hazards such as floods, GLOFs, and landslides have sustained negative impacts on the livelihoods and health of people in the HKH region. Since these disasters cause major damage to property and agricultural lands, people are stripped of their sustenance and source of income. Besides obvious effects of this such as lack of proper food and resources, psychological stress from these events can also have negative impacts on health (Eriksson et al., 2008).

The effects of “slow-onset” disasters, although less conspicuous, are no less devastating than those of “rapid-onset” disasters. Climate change has affected the hydrological cycles, increasing water stresses, water-related illness. In Yunnan province of China, despite the high economic growth in the region in the past 15 years, the rate of water-borne diseases has not declined. Climate change also causes changes in the ecosystem and biodiversity altering people’s diets, causing issues related to nutrition. In Nepal, there is some preliminary evidence that malnutrition in some indigenous groups can be attributed to “diminishing of wild food,” as local flora and fauna are part of their livelihoods (Eriksson et al., 2008).

#### Adaptation and Mitigation Measures Against Climate Change Disasters

Communities in the mountains are more vulnerable to climate change due to several factors including lack of life supporting infrastructure, dearth of government extension agencies, and support services. Lack of support and access make adaptation to climate change difficult for these communities, especially if they include poor and marginalized persons due to socioeconomic inequalities assessment. An assessment of adaptation capacity and measure of communities in the HKH region was conducted by Mishra et al. (2019). They found that there was an urgent need to increase the region’s adaptation capacity, deemed a complex challenge by policy-makers. Adaptation measures for mountain regions have not been integrated well into government plans of actions, as these areas continue to experience lack of information, resources, and adaptation options. The authors posit that it is important to understand and focus on the “autonomous responses” to climate change in these areas, yet the action of subnational levels of government appears inadequate. However, they argue that opportunities such as increasing climate literacy, promoting cooperation among HKH countries and augmented private sector involvement do exist in these areas. To seize these opportunities, an increase in government and private sector funding is essential (Mishra et al., 2019).

### Biodiversity and Health in the HKH Region

#### Effects of Climate Change on Biodiversity and Health in the HKH Region

The HKH region is considered one of the richest biodiversity in the world, harboring several Global Biodiversity Hotspots. The ecosystem and biodiversity of these regions are threatened by human activities such as overgrazing, over-harvesting, pollution, invasive alien species, land use change as well as the effects of climate change (Sharma et al., 2008). These activities have not only diminished natural buffers against emerging infectious diseases but also facilitated dispersal and establishment of species such as invasive alien species, disease vectors that have a negative impact on human health (Shrestha and Shrestha, 2019). It has been predicted that in the next few decades, atmospheric CO_2_ levels will continue to increase and substantially affect the nutrition quality of food. Additionally, climate change is expected to drive changes in species distribution, their abundance, seasonal cycles, disruption of ecosystems, consequently affecting food availability, agriculture patterns, and disease vectors (Romanelli et al., 2015). In Nepal, invasive alien species that have an impact on health, agriculture, and livelihood are expected to increase in future with climate change (Shrestha and Shrestha, 2019). Vulnerable communities like in the HKH region, where populations are not well-equipped to handle these stresses, biodiversity, and ecosystem shifts pose major health challenges.

#### Adaptation and Mitigation Measures on Biodiversity and Health in the HKH Region

Climate change is expected to have a negative impact on biodiversity including medical plants and traditional healing practices and food security thereby increasing the vulnerability of the mountain people who mostly rely on traditional medicine systems. Finally, the impacts of climate change on natural resources, biodiversity, and labor productivity are likely to reduce economic growth, exacerbating poverty through reduced income opportunities (CBD Secretariat, 2009). Furthermore, climate change has also threatened biodiversity and the continued provision of ecosystem services which demands an urgent call for additional research and action toward reducing the impacts of climate change on biodiversity and increasing synergy of biodiversity conservation with climate change mitigation and adaptation activities.

### Climate Change, Gender, and Health in the HKH Region

Climatic change affects people differently according to their socio-cultural, economic and geographical contexts and impacts of climate change are not gender neutral. Although men and women face similar environmental exposures, e.g., ambient air pollution, unsafe water, and noise, many women and small children are exposed to household air pollution at higher levels than men due to their longer working hours around the cook stove (WHO, 2014). It has also been argued that accounting the gender dimensions of climate change adaptation and mitigation would facilitate understanding the root causes of climate change, and to address it in a more sustainable way than is possible with a gender-neutral approach (MacGregor, 2009). Furthermore, climate change vulnerability and adaptive capacity are dynamic in nature, and changes affecting them at one level can have intense and unseen implications at other levels (Pelling, 2010). Hence, failure to integrate gender dimensions for exploring the root causes of vulnerability and finding a sustainable solution might exacerbate rather than reduce injustices, and climate change challenges might remain unaddressed (MacGregor, 2009). The impact of climate change will not be evenly distributed across the geographical regions and socio-economic groups with greater impact among higher vulnerable regions and populations (IPCC, 2007). For instance, a review of 45 case studies from developing and developed countries supported the assumption that climate impacts affect men and women differently, and women tend to suffer more in terms of their assets and well-being (Goh, 2012). Some other literature related to climate change has shown that women will be more vulnerable than men to the effects of climate change because of unequal power relations, limited access to resources (financial, natural, social, and human) and economic opportunities (Denton, 2002; Skutsch, 2002). Some research showed that direct and indirect impacts of climate change and health risks vary for men and women due to their different gender roles (Duncan, 2007).

A study conducted in central Nepal has shown that the prevalence of undernutrition (measured in terms of underweight and stunting) is higher among boys than girls, which was not attributed to any specific reason in the study; however, it suggested that favoritism of boy child which is common in the country might not be significant in the studied community. Additionally, the study found that the education level of mothers seems to be relevant to help reduce the burden of malnutrition (Sarki et al., 2016). Another study from Humla district of northern Nepal (Himalayan region) has shown that the same social and power relations that are governing the local vulnerability dynamics (such as caste, gender, and access to social and political networks), also play vital roles in determining the impact of climate change adaptation policies (Nagoda and Nightingale, 2017). The gender differentiated health impacts of climate change in highland and lowland Nepal is reported (Dhimal, 2018). Women in general, and those living in resource constrained countries in particular, are more vulnerable to the impacts of climate induced disasters (MOHA, 2015).

The impacts of climate change in the forestry and biodiversity sectors directly affect forest dependent households and communities the most, especially the livelihoods of indigenous communities, women, marginalized, and poor people who depend on medicinal herbs and other non-timber forest products for their incomes and livelihoods (MOSTE, 2014; MOPE, 2017). For example, depletion of forest resources would increase the burden on women to gather fuel wood, food, fodder, and medicinal plants (MOSTE, 2014). In Nepal, traditions make women responsible for collecting firewood for cooking, boiling water, and heating rooms. The steep terrain coupled with climate change, which dries up water sources increases the burden and dangers of carrying water, firewood, and fodder, which takes a toll on health (Leduce, 2008, 2009). In addition, indoor smoke from the kitchen affects women and their children more than men, causing respiratory illness (Subha, 1999).

In order to reduce these burdens and improve health conditions as well as reduce greenhouse gases, environmentally friendly technologies such as biogas, improved cooking stoves, and solar cookers need to be introduced in a gender-friendly way. The disruption of ecosystem services such as the availability of safe water, and declines in agricultural productivity also increase the workload of women because the majority of women in the HKH region rely on the agriculture sector, and they are also responsible for fetching water for domestic purposes. For example, in the hills of Sankhuwasabha district in eastern Nepal, the shortage of water is becoming a critical issue, but men tend to qualify the shortage as severe, whereas women tend to qualify the same problem as moderate (ICIMOD, 2009). These differences highlight gendered prioritization since men often consider water shortages for irrigation as a serious problem, while women consider household water access for drinking, cooking food, and sanitation as a serious problem (ICIMOD, 2009). Water shortages disproportionately impose health risks and acute labor burdens on women and girls primarily responsible for collecting and carrying water over long distances across difficult mountain terrain.

#### Adaptation and Mitigation Measures on Gender and Health in the HKH Region

The impacts of climate change are not gender neutral. Accounting for gender in medium- and long-term adaptation plans can help ensure that (1) adaptation is effective and implementable on the ground; (2) gender-specific impacts of climate change are alleviated; (3) implementation of adaptation activities does not exacerbate inequalities and other vulnerabilities in mountain regions (Dazé and Dekens, 2017); and (4) men and women participate equally in the decision-making. Gender-specific differences in vulnerabilities arise due to variations in roles and responsibilities associated with these gender groups and gender-specific vulnerabilities are exacerbated by lack of access to natural, financial, and social capital. Accordingly, targeted policies that seek to address these underlying issues of access to or availability of capitals must be built in climate hotspots such as the HKH Region (Sharma et al., 2019).

## Discussion

This article highlights climate change impacts broadly on environment, safe drinking water, sufficient food, and secure shelter in the HKH region and the consequent effects specifically on vector-, water-, and food-borne infectious diseases, malnutrition, NCDs, and mental health including a gender health perspective. The HKH region is experiencing rapid climate changes (disproportionate increase in temperature in high mountains, reduced snowfall, and extreme weather events including heavy rainfall), which cause a number of socio-environmental changes (increased risk of landslides and other natural disasters resulting from the outburst of glacial lakes, disrupted ground and surface water extraction, the contamination of drinking water sources due to the disruptions of sanitary and sewage systems, altered household, agricultural, and farming productivities in mountain slopes, increased length of season allowing tourist activities, and declining mountain biodiversity). Based on recent literature, there is strong evidence for impacts of climate induced alterations on physical and mental well-being of HKH populations. Climate-sensitive infectious diseases such as dengue virus and malaria-causing *Plasmodium vivax* will expand in HKH regions and the transmission and onset of water- and food-borne illness (cholera, infective gastroenteritis) and an increase in Years Lived with Disabilities (YLDs; bacillary dysentery) will occur at higher elevations. Extreme temperatures associated with increased risk of cardio-pulmonary mortality will occur more frequently. We expect a generally higher health burden for women and children who will suffer more from malnutrition and harder-to-access water and wooden resources and have less power to adapt and mitigate the effects of climate change on various health aspects. The increased frequency of climate induced disasters will further exacerbate the situation.

We proposed adaptation and mitigation measures on human health for the population in the HKH region. A common call is the development and implementation of concerted and sustainable plans to manage the climate, socio-environmental changes at global, national, and subnational levels and program counteracting the lack of information, resources, and adaptation options, especially for hard-to-reach populations such as women, children, and indigenous people in remote areas. We conclude that environment profoundly impacts health, and inter-sectoral collaboration is therefore needed. Nepal’s response to address health impacts of climate change through development of policy and plans is an exemplary (Dhimal et al., 2017). Such action plans are an extremely useful integrative tool to target climate-sensitive diseases and risk. Their implementation is an immense but realizable challenge which, evidently, can only progress if the diversity of gender, populations, environmental threats, and innovative solutions are taken into account. Such a participatory process including the empowerment of the youth, women, and indigenous communities, a sustainable collaboration of HKH countries and the multisectoral implementation of action plans is required to efficiently manage the manifold climate change effects on health and improve the well-being of people in the HKH region.

## Author Contributions

MD: conception, design, and writing. DB, MLD, NK, PP, NM, SA, TI, RD, DG, US, and RM: writing. MD, DB, US, DG, and RM: editing. All authors contributed to the article and approved the submitted version.

## Conflict of Interest

The authors declare that the research was conducted in the absence of any commercial or financial relationships that could be construed as a potential conflict of interest.

## Publisher’s Note

All claims expressed in this article are solely those of the authors and do not necessarily represent those of their affiliated organizations, or those of the publisher, the editors and the reviewers. Any product that may be evaluated in this article, or claim that may be made by its manufacturer, is not guaranteed or endorsed by the publisher.
